# Principles of synaptic encoding of brainstem circadian rhythms

**DOI:** 10.1113/EP090867

**Published:** 2024-02-03

**Authors:** Forrest J. Ragozzino, Ilia N. Karatsoreos, James H. Peters

**Affiliations:** ^1^ Department of Integrative Physiology and Neuroscience, College of Veterinary Medicine Washington State University Pullman Washington USA; ^2^ Department of Psychological and Brain Sciences University of Massachusetts Amherst Amherst Massachusetts USA

**Keywords:** autonomic, diurnal, glutamate, NTS, throughput, vagus

## Abstract

Circadian regulation of autonomic tone and reflex pathways pairs physiological processes with the daily light cycle. However, the underlying mechanisms mediating these changes on autonomic neurocircuitry are only beginning to be understood. The brainstem nucleus of the solitary tract (NTS) and adjacent nuclei, including the area postrema and dorsal motor nucleus of the vagus, are key candidates for rhythmic control of some aspects of the autonomic nervous system. Recent findings have contributed to a working model of circadian regulation in the brainstem which manifests from the transcriptional, to synaptic, to circuit levels of organization. Vagal afferent neurons and the NTS possess rhythmic clock gene expression, rhythmic action potential firing, and our recent findings demonstrate rhythmic spontaneous glutamate release. In addition, postsynaptic conductances also vary across the day producing subtle changes in membrane depolarization which govern synaptic efficacy. Together these coordinated pre‐ and postsynaptic changes provide nuanced control of synaptic transmission across the day to tune the sensitivity of primary afferent input and likely govern reflex output. Further, given the important role for the brainstem in integrating cues such as feeding, cardiovascular function and temperature, it may also be an underappreciated locus in mediating the effects of such non‐photic entraining cues. This short review focuses on the neurophysiological principles that govern NTS synaptic transmission and how circadian rhythms impacted them across the day.

## Abstract (highlights)

- **What is the topic of this review?**
  Synaptic encoding of brainstem circadian rhythms.
- **What advances does it highlight?**
  Rhythmic changes in fast neurotransmission span the time frames between transcription/translation and physiological/behavioural responses. Coordinated pre‐ and postsynaptic neurophysiological changes provide nuanced circadian control to synaptic throughput. Spontaneous vesicle release is a unique cellular mechanism for encoding circadian changes to synaptic and circuit function.

## 1 INTRODUCTION

Circadian rhythms are endogenously generated by the hypothalamic suprachiasmatic nucleus (SCN), and enable the pairing of physiological and behavioural processes with predictable changes in the environmental light cycle caused by the solar day (Buijs et al., 2006; Karatsoreos, 2019). At the cellular level, circadian rhythms are largely driven by a transcriptional–translational feedback loop (TTFL) that serves as a ‘molecular clock’. This pattern of transcription–translation takes approximately 24 h to complete, and regulates downstream cellular processes, including proteins key to neuronal excitability and synaptic transmission (Brown & Piggins, 2007; Colwell, 2000; Dunlap, 1999; Goode et al., 2022; Kentish et al., 2013). While the SCN is the central clock, many other brain areas show clear circadian oscillatory patterns (Begemann et al., 2020), though these rhythms are not sustained without input from the SCN (or external cues). Although in many instances the functional significance of these rhythms remains elusive, rhythms in neural and peripheral tissues responsible for nutrient intake and processing suggest circadian regulation across the neural axis (Chrobok, Ahern et al., 2022; Segers & Depoortere, 2021; Page, 2021).

The brainstem nucleus of the solitary tract (NTS) provides a critical point of control over the autonomic nervous system, including neural coordination of the cardiovascular, respiratory and gastrointestinal systems (Andresen et al., 2004; Clyburn & Browning, 2021), with extensive work detailing the role(s) of the NTS in feeding and satiety (Grill & Hayes, 2012). The NTS receives direct vagal afferent innervation from most visceral organ systems, including the cardiovascular, respiratory and gastrointestinal systems; is responsive to circulating endocrine signals and nutrients; and is thus ideally positioned to coordinate responses to both circadian and homeostatic signals (Chrobok, Ahern, et al., 2022). In rodents, food intake shows strong circadian regulation with the greatest amount eaten at night. Even mild circadian desynchrony results in disrupted timing of feeding and a progression towards obesity (Karatsoreos et al., 2011) and changes in vagal afferent sensitivity to gut stimuli (Page et al., 2020; Salgado‐Delgado et al., 2023). From these findings and others (Chrobok et al., 2020, 2021; Chrobok, Ahern, et al., 2022; Chrobok, Klich, et al., 2022), it is becoming clear that circadian rhythms regulate NTS and vagal afferent circuit function to control the timing of food intake and sensitivity to vagally mediated satiety signals. Further, changes in cardiovascular and respiratory drive, including the autonomic readout of parasympathetic tone and heart rate variability, show circadian rhythmicity that is likely mediated in part via the brainstem changes in synaptic transmission (Singh et al., 2003). However, the mechanisms through which circadian rhythms translate to functional changes in neuronal signalling are only beginning to be understood. Here we focus on several key aspects of this important question with specific emphasis on circadian changes to glutamatergic synaptic transmission with an emphasis on some of our recent findings (Ragozzino et al., 2023).

## 2 PHYSIOLOGICAL TO CELLULAR RHYTHMS

Ultimately, circadian changes in protein expression and other cellular signals must manifest functionally in the nervous system at the level of action potential firing and synaptic transmission to meaningfully alter physiological and behavioural processes. While circadian regulation of genetic/protein level changes as well as behavioural level changes are well documented, relatively few studies have examined the impact of these changes on fast neuronal signalling. Chrobok and colleagues have recently characterized circadian rhythmicity in the dorsal vagal complex (including the NTS), confirming previous descriptions of oscillatory clock gene expression, and extending our understanding to functional changes in blood–brain barrier permeability, action potential firing at the level of multi‐electrode arrays and responsivity to key feeding hormones (Chrobok et al., 2020, 2021; Chrobok, Klich, et al., 2022). This work has significantly extended the conversation around brainstem rhythmicity and provided levels of functional analysis that have not been previously reported in these areas. However, these previous reports have largely focused on intrinsic properties of brain nuclei and have not directly examined the major sensory afferent innervation via the facial, glossopharyngeal, and vagal afferent cranial nerves contained in the solitary tract bundle. Although a recent model incorporates afferent signalling (Chrobok, Ahern, et al., 2022), there remains much to be understood about afferent to NTS signalling in circadian timing. This is important since many ‘non‐photic’ circadian entraining signals are likely to be processed via the NTS, including food availability or changes induced by physical activity. Because of the missing focus on the sensory afferent input and mechanistic neurophysiological studies, our main focus has been to investigate the organization of different forms of synaptic vesicle release and their nuanced control over neuronal and circuit function across the circadian cycle (Ragozzino et al., 2023).

Fast neurotransmission, including the release of glutamate, is governed by vesicle‐mediated exocytosis and is considered ‘quantal’ in nature, with the vesicle representing the minimum unit of synaptic transmission (Katz, 1971). The most commonly considered form of neurotransmission is the action potential‐synchronized release of many vesicles precisely timed with terminal depolarization (Gundersen, 2020). This ‘synchronous’ release is often robust and typically considered the dominant form of signalling. Yet, all synapses possess an alternative vesicle release pathway that is independent of action potential firing (Kavalali, 2020). This action potential‐independent pathway provides a constitutive release of individual vesicles ‘spontaneously’ over time. While previously not regarded as a major contributor to synaptic communication due to its quantal nature, this form of release has become recognized as a major source of synaptic information and point of regulation (Kavalali, 2015). In particular, in the brainstem viscerosensory afferents of the vagus nerve possess prominent spontaneous vesicle release that impacts neuronal signalling and is controlled via many physiological signals, including hormones (Peters et al., 2008), neuropeptides (Appleyard et al., 2005) and temperature (Shoudai et al., 2010). In addition, our recent report demonstrates that spontaneous glutamate release is under circadian regulation and serves to encode time‐of‐day information at the neurophysiological level (Ragozzino et al., 2023).

The spontaneous glutamate release pathway provides a unique advantage to encoding circadian rhythms. Unlike synchronous release, which is bounded by the number of vesicles docked and primed at the membrane and then number of release sites (Neher & Brose, 2018), spontaneous glutamate release has an enormous range of signalling capacity (Kavalali, 2015). Frequency of spontaneous release can scale many orders of magnitude (from 0.1 to 100 Hz) and enables a wide dynamic range. In contrast, under physiological conditions, synchronous vesicle release at vagal afferent to NTS synapses typically only scales by a doubling or less (Wu et al., 2014).

Changes to the spontaneous release pathway, including circadian control, are largely governed presynaptically via targeting the probability of vesicle release. The probability of release is dependent on presynaptic calcium and its interactions with the key calcium binding protein synaptotagmin (and its many forms, synaptotagmins 1–7 (Sudhof, 2012). Changes in calcium concentrations, buffering or the vesicular binding proteins can shift the probability of release (Catterall, 1999; Peters et al., 2010, 2008)). Given the prominence of spontaneous release in the NTS from the vagal afferent neurons, even small shifts in release can produce large changes in signalling over circadian time scales (Ragozzino et al., 2023). However, these presynaptic changes in transmitter release are realized via their actions on postsynaptic receptors and the filter of the postsynaptic membrane resistance.

## 3 COORDINATE POSTSYNAPTIC CHANGES

Synaptic throughput is governed by both pre‐ and postsynaptic variables (Silver, 2010). The postsynaptic membrane resistance determines the voltage change produced for a given current injection (synaptic event) as dictated by Ohm's law. Membranes with higher electrical resistance (tighter) produce larger voltage changes compared to membranes with lower resistance (leakier). Thus, subtle changes in the postsynaptic membrane resistance, through the closing or opening of ion channels (typically background sodium (Simasko, 1994) and potassium channels (Roberts & Karatsoreos, 2023)) could provide an important point of control to synaptic throughput. In the NTS, we found a seemingly paradoxical change in synaptic throughput across the circadian day (Ragozzino et al., 2023). Whereas the frequency of spontaneous release was highest during the light, the action potential‐driven throughput was lowest. The opposite was true during the dark, where the action potential‐driven synaptic throughput was highest, even though spontaneous glutamate release was at a nadir. The seemingly opposing circadian effects on pre‐ and postsynaptic properties enable both predictive and responsive signalling tuned to the time of day and physiological demand. In this model, viscerosensory information is diminished during the inactive light phase, when the animal is typically not eating or moving as much. During this period the increased spontaneous glutamate release and subsequent increase in basal action potential firing likely mediate a satiated, rest‐and‐digest state at the level of the NTS and brainstem. In contrast, during the active dark phase, spontaneous glutamate release is low, basal action potential firing is low, and the postsynaptic membrane has a higher resistance and thus is more responsive to incoming vagal signals. This positions the synapse to be more responsive to incoming vagal signals to coordinate feeding and other autonomic reflex pathways with fidelity (Ragozzino et al., 2023).

The mechanisms of the postsynaptic changes in membrane potential and how it is coordinated with the presynaptic release remain unknown. The reported change in conductance is consistent with increasing and decreasing a background sodium conductance (Ragozzino et al., 2023), as this mechanism has been observed before with changes in the open probability of background sodium channels that encode rhythmicity and burst firing into excitable cells and neurons (Simasko, 1994). We speculate in our recent paper that this is likely the same mechanism seen in the NTS neurons (Ragozzino et al., 2023). This rhythmicity may arise via cell autonomous TTFL control of transcription or other regulatory enzymes which gate the conductance. Alternatively, the degree of spontaneous synaptic input may also inform the timing and gating of these channels and subsequent membrane resistance. Experiments aimed at delineating the postsynaptic mechanisms of the rhythmic conductance will need to isolate the role of synaptic input, perhaps via promoting or inhibiting glutamatergic signalling or by recording NTS neurons not directly innervated by the vagus. If similar to other circadian processes, there is a good chance of both TTFL and activity‐driven signals coordinating the change and setting the timing across the day.

## 4 HORMONES, CALCIUM AND TIME KEEPING

Given the importance of the spontaneous release pathway, it should not be surprising that many hormones (including those that control gastrointestinal and autonomic function) target this form of signalling (Appleyard et al., 2005; Cui et al., 2011; Peters et al., 2008; Williams & Smith, 2006). Most paracrine and endocrine signals, including the satiety peptide cholecystokinin, utilize cognate receptors that are G‐protein coupled receptors which couple via either membrane‐bound ion channels to permit extracellular influx of calcium (Kowalski et al., 2020), or though the release of intracellular calcium stores (Gonzalez et al., 1999), or both pathways to control the spontaneous release of glutamate. In the NTS, the central terminals of vagal afferent neurons express many transient receptor potential (TRP) ion channels, including the canonical capsaicin receptor (TRPV1) (Andresen, 2019). Activation of these TRP channels selectively targets the spontaneous vesicle release pathway, and thus their coupling to the hormone G‐protein coupled receptors ensures that they prominently impact spontaneous release over other forms of synaptic transmission.

While the common mechanism of action between hormonal and circadian rhythms, specifically the spontaneous glutamate pathway, suggests crosstalk between these physiological signals, what remains only partially understood is the extent to which hormone signalling in the vagus and brainstem serve as time givers and tune the circadian clock (Chrobok, Ahern, et al., 2022; Chrobok, Klich, et al., 2022). Certainly, timing of feeding can inform central time keeping; however, it is not clear the gut‐derived hormones themselves alter the TTFL or if they superimpose their signalling on the intrinsic and SCN circadian rhythmicity (Salgado‐Delgado et al., 2023). Careful experiments directly testing the ability of hormones that signal via the vagus and NTS to set the circadian timing are needed and will be enlightening as to the hierarchy of circadian time givers in the brainstem.

## 5 CIRCADIAN SYNAPTIC CHANGES ACROSS AUTONOMIC SYSTEMS

All autonomic reflexes and responses show diurnal variation and are generally considered to have some aspect of circadian regulation. However, the degree to which circadian changes in primary vagal afferent to NTS synaptic release of glutamate impact specific autonomic processes remains to be determined. So far, most of our theorizing has been in the context of feeding and saliency of satiety signals and gastrointestinal responses. However, it is also important to consider how these changes may impact cardiovascular and respiratory physiology across the circadian day. It seems reasonable that the observed changes in action potential‐driven synchronous glutamate release have good explanatory power for the marked circadian changes in baroreflex function (Hossmann et al., 1980; van Eekelen et al., 2004). Similarly, it is well documented that respiratory drive is diminished during the rest phase, consistent with the reduced afferent signalling (Raschke & Moller, 1989). While causal relationships between the circadian changes in vesicle release and these physiological changes should be tested, it is clear that circadian desynchrony (for example, chronic rotating shift work) greatly increases the risk of cardiorespiratory issues and related mortality. These pathophysiologies of timing may be in part mediated via changes in central synaptic signalling from the primary afferents to NTS, thus suggesting a potential point of intervention.

## 6 CONCLUSIONS AND MOVING FORWARD

Brainstem circadian rhythmicity is a fundamental attribute that facilitates efficient and appropriate autonomic and behavioural responses to daily feeding and other physiological challenges. It may also contribute to mechanisms by which non‐photic cues are able to synchronize circadian oscillators throughout the brain and body, sometimes without an impact on the SCN clock directly. The model of coordinated pre‐ and postsynaptic responses, in particular the targeting of the spontaneous glutamate release pathway, may be a common mechanism observed at other synapses exhibiting circadian rhythmicity. The ubiquity of the spontaneous release pathway and its responsiveness to many physiological and hormonal signals positions this mechanism as a great integrator of both photic and non‐photic timekeepers. While plausible, much experimental work is still needed to fully understand the organization and mechanism translating clock gene expression to functional neurophysiological outputs.

## AUTHOR CONTRIBUTIONS

Forrest J. Ragozzino, Ilia N. Karatsoreos and James H. Peters conceptualized the review. Forrest J. Ragozzino and James H. Peters wrote the first draft of the manuscript. Forrest J. Ragozzino, Ilia N. Karatsoreos and James H. Peters edited the draft. All authors have read and approved the final version of this manuscript and agree to be accountable for all aspects of the work in ensuring that questions related to the accuracy or integrity of any part of the work are appropriately investigated and resolved. All persons designated as authors qualify for authorship, and all those who qualify for authorship are listed.

## CONFLICT OF INTEREST

No competing interests declared.
